# Nile Tilapia Skin Xenograft Versus Silver-Based Dressings in the Management of Partial-Thickness Burn Wounds: A Systematic Review and Meta-Analysis

**DOI:** 10.3390/jcm13061642

**Published:** 2024-03-13

**Authors:** Francisco Cezar Aquino de Moraes, Bárbara Ferraz Barbosa, Debora Sepulvida, Camila Bordignon Barbosa, Luiza Miziara Brochi, Edmy Soza Figueroa, Marianne Rodrigues Fernandes, Ney Pereira Carneiro dos Santos

**Affiliations:** 1 Oncology Research Center, Federal University of Pará, Belém 66073-005, PA, Brazil; 2 Department of Medicine, University of Aquino Bolivia, Santa Cruz de la Sierra 0701, SC, Boliviabordignoncamila4@gmail.com (C.B.B.); 3 Department of Medicine, Tufts Medical Center, Boston, MA 02111, USA; 4 Department of Medicine, University of Uberaba, Uberaba 38055-500, MG, Brazil; 5 Plastic and Reconstructive Surgery Service, Department of Pediatrics, Maternal and Child Hospital, Santa Cruz de la Sierra 0701, SC, Bolivia

**Keywords:** tilapia skin, burns, sulfadiazine, meta-analysis

## Abstract

**Background**: Burns are a serious public health problem worldwide, causing high morbidity and mortality. This study aimed to compare two forms of treatment for partial skin burns and to determine whether one is superior to the other in terms of efficacy and benefits through a meta-analysis of randomized controlled trials. This article highlights the efficacy of tilapia skin in the treatment of burns. We performed a meta-analysis of 199 patients and highlighted the promising results that indicate the clinical relevance of this resource when we compared the cost of dressings with the daily need for dressing changes, healing potential, and reduction in pain level according to the VAS scale and reduced frequency of dressing changes. **Methods**: A search of PubMed, Cochrane Central, and LILACS was performed to identify randomized controlled trials comparing tilapia skin and silver-based dressings for treating burns. Studies involving overlapping populations and animals were excluded. The outcomes of interest were complete re-epithelialization, decreased pain level, and dressing change. **Results**: Summarize the article’s main findings. **Conclusions**: Four randomized trials were included with a total of 199 patients with partial-thickness burns between the ages of 2 and 70 years. A total of 99 (49.74%) patients were treated with tilapia skin, and conventional treatment was used on 100 (50.25%) of the patients. Differences were found between the tilapia and silver-based treatments concerning re-epithelialization (MD −0.48; CI 95% −0.71 to −0.24; *p* < 0.01; I2 = 0%), decreased pain level (MD −0.79; CI 95% −1.10 to −0.47; *p* < 0.01; I2 = 0%), and dressing change outcome (MD −3.54; 95% CI −5.81 to −1.26; *p* = 0.02; I2 = 97%).

## 1. Introduction

Every year, several people die from burn injuries worldwide. In addition, nonfatal burn victims may be subjected to several complications, such as pain, dehydration, tissue changes, patient disfigurement, prolonged hospitalization, infections, and social stigma [1]. The treatment of severe burns deserves special attention and should be conducted in an in-hospital environment by a multidisciplinary team [2]. The usual therapeutic approach consists of using silver-based creams and dressings, such as 1% sulfadiazine, which have been used since the 18th century [3]. At the same time, severe burns require more complex approaches, as full-thickness wounds need biomaterials, synthetic dressings, and human skin allografts or xenografts to be effectively repaired [4,5]. The poor availability and high cost associated with human skin allografts has been driving the research of xenografts derived from other materials [3].

More recently, tilapia skin (a fish from the Nile River basin) has been widely studied as a possible resource for the treatment of burns. It is an alternative to frog and porcine skins, which, despite promising results in previous research, have some disadvantages, such as the high cost and low availability in the market [6,7]. The advantages of using tilapia skin, in addition to its low cost, are associated with its biological potential in covering burns, as it is a flexible material with good adhesion to the tissue, a large amount of collagen I in its composition, and histopathological and morphologic similarity to human skin, as well as the presence of noninfectious microbiota after cleaning procedures and material preparation [8,9].

Despite promising previous studies with tilapia skin, there is still no consensus on its clinical benefits in relation to outcomes such as pain, improved healing, and reduction in the number of dressings. Therefore, the aim of this study is to compare tilapia skin xenografts to silver-based dressings as treatment methods for partial skin burns, focusing on these clinical outcomes.

## 2. Materials and Methods

In this section, we outline the eligibility criteria, search strategy, data extraction methods, endpoints, subgroup analyses, quality assessment, and statistical analyses employed in our study.

### 2.1. Eligibility Criteria

Inclusion in this meta-analysis was restricted to studies that met all the following eligibility criteria: (1) randomized controlled trials (RCTs); (2) comparing Nile tilapia skin xenografts with silver-based dressings; (3) patients treated for burn wounds; and (4) reporting clinical outcomes, including complete re-epithelialization per number of days and pain intensity assessed with VAS and the revised faces pain scale (FPSS-R). We excluded studies with (1) meta-analyses and literature reviews; (2) animal studies; (3) conference abstracts and incompletely reported studies; and (4) overlapping patient populations. In the last case, only the study with the highest number of patients was included. There was no restriction concerning the age or sex of the patients involved, the thickness of present burns, or the body surface burned.

### 2.2. Search Strategy and Data Extraction

The search was performed via PubMed, the Cochrane Central Register of Controlled Trials, and LILACS for studies that met the published eligibility criteria from inception through October 2022. The search strategy was based on keywords such as “tilapia”, “tilapia skin”, “burns”, and “burn” using the Boolean operators “AND” and “OR”, which were adapted to the syntax rules of each platform. In addition to the database search, the references of the included studies were manually reviewed. There were no restrictions on the language of the articles. The search was conducted by two independent authors (F.B and F.M) following predefined research criteria in the inclusion and exclusion criteria. Disagreements were resolved by a third author (D.S). The prospective meta-analysis protocol was registered in PROSPERO 2022, CRD42022369475.

### 2.3. Endpoints and Subgroup Analyses

Efficacy outcomes included (1) complete re-epithelialization by a number of days; (2) pain intensity; and (3) the number of dressings performed. The definitions of complete re-epithelialization did not vary in the different studies, and healing was considered complete at ≥95% of re-epithelialization.

### 2.4. Quality Assessment

A quality assessment of randomized controlled trials (RCTs) was performed with Cochrane’s tool [10] for assessing bias, wherein studies are scored as high, low, or unclear risk of bias in 5 domains: selection, performance, detection, attrition, and reporting [11]. The risk of bias assessment was the tool of choice. It was performed by two independent authors (F.B. and S.D.). Disagreements were resolved by consensus after discussing the reasons for the divergence. Publication bias was investigated with a funnel plot analysis of the primary outcomes.

### 2.5. Statistical Analyses

This systematic review and meta-analysis were performed under the recommendations of the Cochrane Collaboration and the guidelines of the Preferred Reporting Items for Systematic Reviews and Meta-Analyses (PRISMA) statement [12]. Mean difference (MD) and standardized mean difference (SMD) with 95% confidence intervals (CI) was used to compare treatment effects with continuous outcomes. Heterogeneity was examined with the Cochran Q test, I2 statistics, and visual inspection of forest plots, and it was considered significant if the *p*-value was less than 0.10, the I2 statistic exceeded 25%, or the visual inspection of the forest plot was indicative of heterogeneity in the effect size. However, the outcome of this review showed visual homogeneity, I2 statistic <25%, and *p* > 0.10, suggesting no heterogeneity; thus, we used a fixed effect model. A statistical analysis was performed using Review Manager 5.4 (Nordic Cochrane Center, The Cochrane Collaboration, Copenhagen, Denmark).

## 3. Results

### 3.1. Study Selection and Characteristics

**Figure 1:**
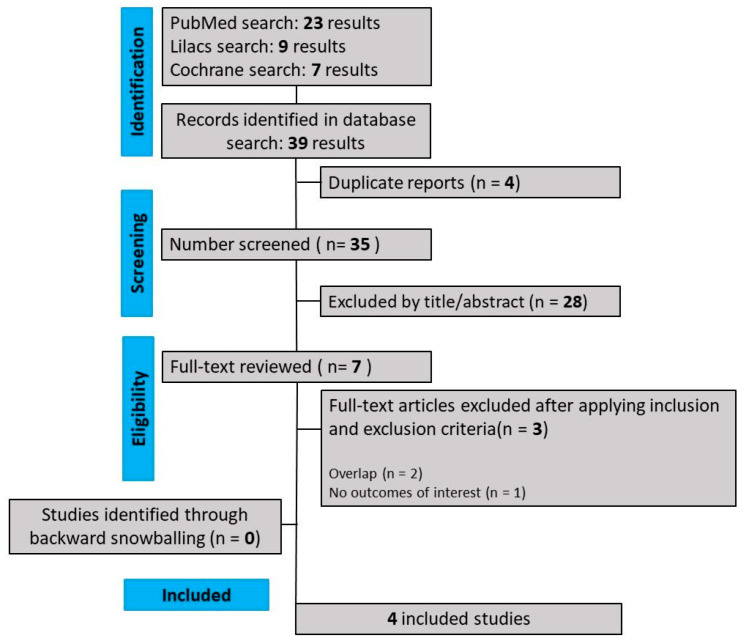
PRISMA flow diagram of study screening and selection.

As detailed in Figure 1, our comprehensive search yielded 441 results, of which 88 were duplicate records, and 346 articles were considered unrelated based on their titles or abstract reviews and were excluded. The remaining seven articles were thoroughly selected, and after applying the inclusion and exclusion criteria, four RCTs were included in this systematic review and meta-analysis. The reasons for study exclusion were overlapping studies (*n* = 3). The main characteristics of individual studies are presented in Table 1.

### 3.2. Demographic and Clinical Data

After thoroughly analyzing each individual study, all 199 patients were included. As shown in Table 1, patients are victims of partial-thickness burns (superficial and deep second degree) aged 2 to 70 years old, with similar clinical and demographic characteristics at baseline for the treatment groups. Of these, 99 (49.74%) patients were randomized to receive treatment with tilapia skin, while the remaining patients underwent treatment with silver-based dressings (50.25%).

For the assessment of the main outcomes, exact data extracted from each study were used in this meta-analysis. Regarding re-epithelialization, the mean and SD of the studies conducted by Lima and Miranda [3,13,14] were used. For pain assessment, the calculation was performed based on SMD. Studies including adult patients [13,15] reported mean and SD based on quantification using VAS. As for the Lima study [14], which included children from 2 to 12 years old, pain was measured using the revised faces pain scale (FPSS-R). The mean and SD of the studies by Lima [11,13,15] were used for the outcome of the number of dressing changes [15].

### 3.3. Pooled Analysis of Outcomes and Subgroup Analyses

This section presents the outcomes of this study, including study selection, demographic and clinical data, pooled analysis of outcomes, and quality assessment.

#### 3.3.1. Re-Epithelialization

**Figure 2:**
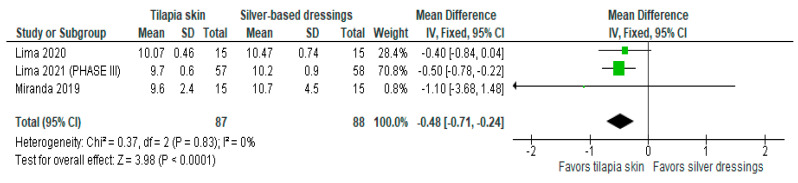
Re-epithelialization was significant in the tilapia skin group. Green squares, studies included in this analysis; black rhomb, combined analysis. In this analysis, we included: Miranda [3], Lima [13] and Lima [14].

Of the four studies [3,11,13,15] that evaluated re-epithelialization, three [3,11,13] used mean and SD to report the data, and one [15] exposed the result imprecisely (10 to 11 days). The three studies were included in the meta-analysis to assess re-epithelialization in burn patients using tilapia skin (Figure 2), the results of which showed a significantly lower mean compared to those treated with silver-based dressings (MD −0.48 (95% CI −0.71, −0.24), *p* value < 0.0001% and heterogeneity verified using the I2 test = 0%).

#### 3.3.2. Pain Intensity

**Figure 3:**
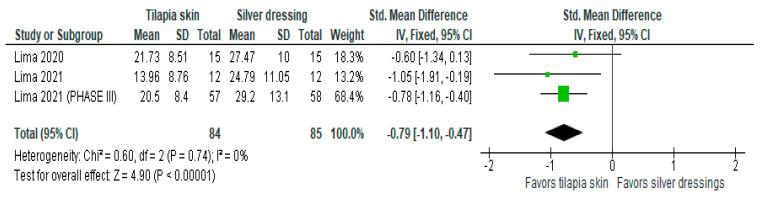
Pain-intensity was significant in the tilapia skin group. Green squares, studies included in this analysis; black rhomb, combined analysis. In this analysis, we included: Lima [13], Lima [14] and Lima [15].

Of the four studies [3,11,13,15] that evaluated the level of pain, three [11,13,15] used mean and SD, and one [3] exposed the result in a number of events (%). Despite the heterogeneity between the scales used as mentioned above, the three studies with continuous values were included in the forest plot that evaluated pain in burn patients with the use of tilapia skin during treatment (Figure 3), the results of which showed improvement in the level of pain intensity when using tilapia skin, compared to silver-based dressings (MD −0.79 (95% CI −1.10, −0.47), *p* value < 0.00001% and heterogeneity verified using the I2 test = 0%).

#### 3.3.3. Number of Dressings Performed

**Figure 4:**
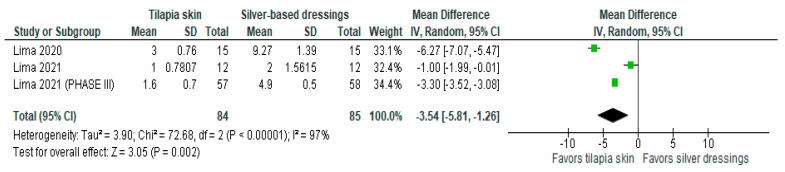
Number of dressings performed was significant in the tilapia skin group. Green squares, studies included in this analysis; black rhomb, combined analysis. In this analysis, we included: Lima [13], Lima [14] and Lima [15].

Of the four studies [3,11,13,15] that evaluated the number of dressing changes, two [11,13] compared Nile tilapia skin with 1% silver sulfadiazine cream, changing dressings in the intervention group if there was a failure in the adhesion to the wound bed and in the control group every 24–48 h. The results were expressed as mean and SD. The results of the study showed less need for dressing changes when tilapia skin was used compared to silver-based dressings (MD −3.54 (95% CI −5.81, −1.26), *p*-value = 0.002% and heterogeneity verified using the I2 = 97%) (Figure 4).

#### 3.3.4. Combined Outcomes

Three studies [11,13,15] provided data regarding the combined results. With 169 patients (Table 2), Lima phase III [13] was the only study to show statistical significance in oral dipyrone intake between the groups (*p* < 0.001), and there was no difference in the use of tramadol in the two studies analyzed. Regarding the assessment of pain-related anxiety, the burns specific pain anxiety scale (BSPAS) scores obtained were significantly lower in the Nile tilapia fish skin group of the phase III study [13] and showed no difference in the phase II study [15]. When compared to the amount of intravenous ketamine used during the procedure, there was a statistically significant difference (*p* = 0.0014) in the induction of anesthesia performed in children [11]. Cost-effectiveness was relatively lower in the tilapia group (*p* < 0.001) [13].

### 3.4. Quality Assessment

**Figure 5:**
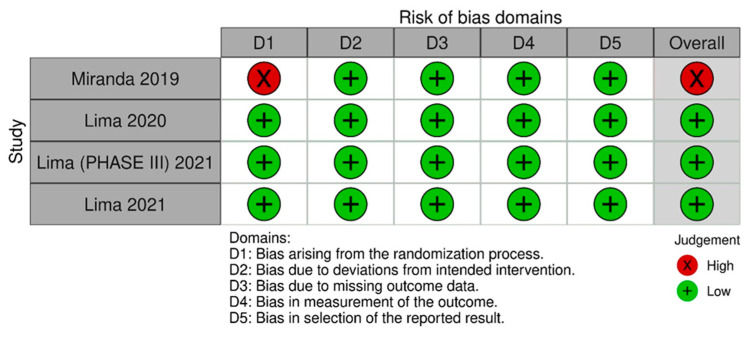
Risk of bias domains 2. In this analysis, we included: Miranda [3], Lima [13], Lima [14] and Lima [15].

Figure 5 [16] summarizes the individual quality assessment of each RCT included in the meta-analysis. Three studies [11,13,15] were rated as having a low risk of bias, while we had some concerns about one [3]. Although a small number of studies were included, the analysis of funnel plots (Figures S1–S3) of the primary outcome showed a symmetric distribution of studies with similar weights and point estimates.

### 3.5. Sensitivity Analysis

Heterogeneity was greatest for dressing changes, which decreased by 2% from baseline after the Lima study [11] was discontinued (I2 = 95%) (MD −2.20; 95% CI −4.45, 0.05; Figure S4).

## 4. Discussion

In this systematic review and meta-analysis of four studies and 199 patients, we compared the use of Nile tilapia skin with the standard treatment performed with silver-based dressings for partial-thickness skin burns, focusing on these clinical outcomes. The main findings were as follows: a statistically significant decrease in the number of days for complete re-epithelialization, a decrease in the level of pain reported by patients during treatment, and less need to change dressings.

For more than four decades (1967), since its introduction into clinical practice by Charles Fox Jr, silver sulfadiazine has been the gold standard for the topical treatment of burns of varying thickness [17,18].

Despite its antibacterial potential, which has potentially improved the chance of survival for patients with severe burns, in the last 10 years, there have been several reports of toxicity, impaired assessment of healing depth and status, and frequent, painful, and challenging dressing changes, concomitant with an expensive and laborious cost–benefit ratio [18]. Due to these disadvantages of SSD and the need for a treatment that provides temporary wound closure along with a moist environment that stimulates healing, numerous biological collagen-based dressings have been used. Some of these include human amnion and dermal matrices (bovine and porcine), which have been discontinued from the market in most countries due to their numerous disadvantages and high cost [19].

A Cochrane review conducted in 2020 with the aim of comparing different topical treatments, including TransCyte, allograft, and porcine xenograft for facial burns, concluded that there was no high-quality evidence to show that a skin substitute could slightly reduce the time to partial wound healing compared to an unspecified antibacterial agent. This conclusion was reached after evaluating wound infections, healing, treatment time and pain at the time. However, some isolated studies showed that TransCyte (skin substitute) could slightly reduce pain (low-quality evidence). In comparison, a biological dressing (Xenoderm porcine) can slightly increase pain in burns compared to antimicrobial agents [20]. It is important to note that the Cochrane review did not directly address tilapia skin.

The NTS is considered a pioneering product of animal origin and is developed in the northeast of Brazil for human use. The skin offers excellent availability and cost-effectiveness and is easy and quick to apply, requiring fewer exchanges, cost of hospitalizations, and workload of the hospital staff, in addition to demonstrating faster healing and better control of reported pain. Histologically, it has a morphological structure similar to that of human skin, with a greater composition of type I collagen and without variations in its characteristic after sterilization procedures. In addition, it demonstrates nontoxicity, as reported in the studies, and demonstrates excellent results for the treatment of thick burns in a pediatric population [11,21].

In the case of Nile Tilapia skin as a xenograft, initially, glycerolization was the preferred method, but with research advances, the possibility of lyophilization was seen so that the skin retained the same clinical efficacy and safety, being implemented in one study [15]. These results were compared with Aquacel Ag^®^ (Convatec, Bridgewater, NJ, USA), a composite occlusive dressing, which is part of current standard care, demonstrating results similar to those of previous research.

In some studies [3,15], pain was compared before and after applying and changing dressings, as well as during daily visits. Lima [15], concluded that there was no statistically significant difference in pain intensity before the procedure (LNTS 10.21 ± 12.54 and NaCMC-Ag 13.96 ± 8.76, *p* = 0.1697). However, post-procedure pain measured using VAS showed a significant decrease, with a mean difference of 10.83 (95% CI, 2.39–19.27, *p* = 0.0142). Lima [11] demonstrated that the change in dressings with the xenograft was significantly lower (3.00 ± 0.76—*p* < 0.0001) compared to the SSD-based cream (9.27 ± 1.39). The Lima 2021 phase III^12^ study also showed a significant reduction of the change in dressings (*p* < 0.001). The other two studies [3,15] performed comparisons between fish skin and a silver-based Hydrofiber dressing (Aquacel Ag^®^). Lima [15] also demonstrated that dressing changes were significantly reduced (*p* < 0.0001), as well as the study by Miranda 2019 [3], which presented its results separated into two groups: sixteen (53.33%) patients who did not undergo any exchange, including nine (60%) in the tilapia group and seven (46.7%) with Aquacel Ag^®^. The second group of study results included fourteen (46.67%) patients requiring one or more changes, including six (40%) patients with tilapia dressings and eight (53.3%) with Aquacel Ag^®^ dressings, having a *p* of 0.71 (*p* ≥ 0.05) and demonstrating the noninferiority of the Nile Tilapia skin dressing compared to Aquacel Ag^®^.

The level of I2 for re-epithelialization and pain assessment was not as significant as it was for dressing changes. It is believed that the main predisposing factor is the large difference in the need for dressing changes in the population treated with silver-based dressings. Therefore, this is a confounding factor to be considered. However, this difference between dressings is expected, and the occurrence of high heterogeneity does not contraindicate the use of tilapia skin. The sensitivity analysis did not indicate which study caused the high heterogeneity of the analyzed results. Also, it is assumed that the inclusion of patients from different age groups (adults and children) in the composition of the study population did not increase the degree of heterogeneity in the results and that the heterogeneity of results might stem from a significant difference in the need for dressing changes in the population treated with silver-based dressings.

The phase III study [13] evaluated the total costs of the dressings used in the treatment with tilapia skin at US $613 (for 57 patients), with a 45.5% reduction in total costs compared to the traditional treatment with silver sulfadiazine cream (US $1123, for 58 patients), which was effectively lower (*p* < 0.001). In terms of analgesic and anesthetic intake, two studies [11,15] did not show statistical significance in the number of milligrams of oral dipyrone (*p* = 0.6969 and *p* = 0.4050, respectively); however, Lima [13] showed statistical significance in oral dipyrone intake between the groups (*p* < 0.001). When compared with the amount of intravenous ketamine used during the anesthetic procedure, a statistically significant difference (*p* = 0.0014) was noted [11]. Anxiety related to pain was measured using the BSPAS scores. The values obtained showed no significant difference (*p* = 0.3100) in the patient’s level of anxiety to painful procedures between the groups [15], showing positive outcomes in the phase III study (*p* = 0.035) [13].

Fish skin grafting is an emerging technique that has gained much notoriety recently [22,23,24,25,26]. A systematic review conducted in 2022 [27] that included 14 studies investigated the effects of acellular fish skin on burns or donor areas. Its conclusions corroborate the evidence of our work. The authors demonstrated an acceleration of wound healing, a reduction in pain and dressing changes required, a reduction in the costs associated with treatment, and better aesthetic and functional results compared to conventional treatment options.

In the study conducted by Cochrane (2020), it was recommended that for the treatment of burns, patient satisfaction or comfort should guide medical decisions and conduct, taking into account a treatment that uses the least need for dressing changes, the shortest time for total healing, reduced pain, and improved quality of care [20].

This is the first meta-analysis to evaluate the use of tilapia skin in burn patients. Nile tilapia skin was compared to silver-based dressings. The main findings suggest that there is a significant difference in the number of days for complete re-epithelialization and a decrease in the patient’s pain levels in comparison to those who received treatment with silver-based dressings, in addition to a decrease in the need to change dressings. The reduction in the need for dressing changes is beneficial for both patients and medical staff. The strength of this meta-analysis was the use of all available published randomized studies. An additional analysis was also conducted to check patients’ tolerance of taking pain-relieving drugs during treatment and surgical procedures, and all outcomes were included. The findings of our meta-analysis strongly suggest that the use of tilapia skin significantly increases healing in this population, which is associated with reduced pain and dressing changes, as well as good tolerability and safety.

## 5. Limitations

The sample size of the population is not a limitation due to the feasibility of the hypothesis and the rarity of treatment for these individuals in the field of plastic and reconstructive surgery. Finally, the age of the patients and the weight of the sample in individual studies may accidentally be a biasing factor that contributes to the increased heterogeneity of the results due to the insufficient information available in some published studies. In the same way, in these studies, it is not possible to conduct blind and double-blind studies due to the need to visualize the treatment to be performed, evaluated, replaced, and removed. Regarding the results in the pediatric population in the individual study, the small sample size could be an important factor in differentiating the results of re-epithelialization or skin regeneration capacity at pediatric age, requiring further randomized studies in the area. There is also a clear limitation related to the lack of randomized studies conducted comparing tilapia skin to other types of more modern treatments. This is because, due to advances in biomolecular technology, countries with greater biotechnological development no longer use SSD cream as a standard treatment due to the high need for daily dressing changes and suboptimal results.

## 6. Conclusions

Evidence supports the efficacy of tilapia skin as a xenograft, which is superior in the treatment of people with partial-thickness (second degree) burns. The use of this treatment directly results in a reduction in treatment/hospital days due to fewer days to complete healing, fewer necessary dressing changes, and a reduction in pain scores and the use of analgesics. The treatment has been well-tolerated with good safety in all studies and is indicated for the short- and long-term treatment of superficial and deep burns in all age groups.

## Figures and Tables

**Table 1:** Demographic and clinical data.

	Lima (Phase III), 2021		Lima, 2021		Lima, 2020		Miranda, 2019	
	Glycerolized Tilapia Skin	SSD	Lyophilized Tilapia Skin (LNTS)	Silver Hydrofiber	Tilapia	SSD	Tilapia Skin	Silver Hydrofiber
Location; Period	Fortaleza/CE, Brazil; April 2017 to October 2018		Fortaleza/CE, Brazil; April 2019 to December 2019		Fortaleza/CE, Brazil; May 2017 to March 2018		Recife/PE, Brazil; 2018	
Number of patients	115		24		30		30	
Sample size	57	58	12	12	15	15	15	15
Sex: -Male -Female	31 (54.4%) 26 (45.6%)	26 (44.8%) 32 (55.2%)	9 (75.00%) 3 (25.00%)	4 (33.33%) 8 (66.67%)	10 (66.6%) 5 (33.3%)	8 (53.3%) 7 (46.6%)	NA	NA
Age (years): mean ± SD	37.1 ± 11.7	39.6 ± 10.1	39.08 ± 12.94	39.33 ± 12.51	5.67 ± 3.66	5.20 ± 2.70	NA	NA
BMI (kg/m^2^): mean ± SD	25.5 ± 7.3	25.0 ± 3.0	3.04 ± 1.54	25.53 ± 1.43	17.75 ± 2.56	18.16 ± 2.74	NA	NA
Percentage of TBSA burned: mean ± SD	2.7 ± 1.8	3.1 ± 2.3	3.04 ± 1.54	2.79 ± 1.48	11.13 ± 4.94	10.13 ± 4.16	NA	NA
Global clinical impression (burn severity): median (IQR)	3 (3–4)	3 (5.2%)	3.00 (3.00–3.00)	3.00 (3.00–3.00)	4 (4–4)	4 (4–4)	NA	NA
Pain intensity using VAS: median (IQR)	3 (3–4)	3 (5.2%)	2.00 (0.25–3.00)	3.50 (1.25–7.25)	9.20 ± 1.47	8.00 ± 3.21	NA	NA
Mechanism of burn -Scald -Flame -Hot surface -Other	50 (87.7%) 4 (7.0%) 4 (7.0%) 2 (3.5%)	2 (3.5%) 6(10.3%) 3 (5.2%) 3 (5.2%)	12 (100%) NA NA NA	12 (100%) NA NA NA	14 (93.33%) 1 (6.67%) NA NA	12 (80%) 3 (20%) NA NA	45% NA NA NA	45% NA NA NA

SSD, Silver sulfadiazine; NA, not evaluated; ±SD, standard deviation. In this analysis, we included: Miranda [3], Lima [13], Lima [14] and Lima [15].

**Table 2:** Combined outcomes.

Study (Years)	Tilapia Skin	Silver-Based Dressings	*p*-Value
**Analgesic Intake**	**Dipyrone**		
**Lima 2021 PHASE III**	1000; range, 500 to 1750 mg	2000 mg; range, 1000 to 3000 mg	*p* < 0.001 *
**Lima 2020**	3561.67 ± 2135.14	3246.67 ± 2247.83	*p* = 0.6969
**Lima 2021**	0.00 (0.00–1000.00)	500.00 (0.00–1000.00)	*p* = 0.4050
**Tramadol**			
**Lima 2021 PHASE III**	-		*p* = 0.872
**Lima 2020**	-		*p* = 0.4049
Pain-related anxiety evaluation *			
**Lima 2021 PHASE III**	5; range, 0 to 15	8; range, 1.75 to 22.25	*p* = 0.035 *
**Lima 2021**	0.00, 0.00–6.75	3.50, 0.00–17.25;	*p* = 0.3100
**Anesthesia**	Intravenous ketamine *		
**Lima 2020**	76.73 ± 39.12	150.07 ± 70.14	*p* = 0.0014 *
Cost-Effectiveness Analysis *			
**Lima 2021 PHASE III**	$11 ± $1 per patient	$19 ± $1 per patient	*p* < 0.001 *

±, standard deviation; * statistically significant. In this analysis, we included: Lima [13] and Lima [14].

## Data Availability

Not applicable.

## Supplementary Materials

The following supporting information can be downloaded at: https://www.mdpi.com/article/10.3390/jcm13061642/s1, Figure S1. Funnel plot of comparison: 2 Tilapia Skin × silver-based dressings, outcome: 3.2.1 Complete re-epithelialization; Figure S2. Funnel plot of comparison: 2 Tilapia Skin × silver-based dressings, outcome: 3.2.2 Pain Intensity; Figure S3. Funnel plot of comparison: 2 Tilapia Skin × silver-based dressings, outcome: 3.2.3 Number of dressings performed; Figure S4. Number of dressings performed leave one out analysis.

## Abbreviations

BSPAS	Burns specific pain anxiety scale
FPSS	Faces pain scale-revised
LNTS	Lyophilized Nile tilapia skin
NaCMC-Ag	Silver-impregnated sodium carboxymethylcellulose
NTS	Nile tilapia skin
PRISMA	Preferred Reporting Items for Systematic Reviews and Meta-Analysis
RCT	Randomized controlled trial
SSD	Silver sulfadiazine
VAS	Visual analog scale
WHO	World Health Organization
