# Supplementary material for: Nile Tilapia Skin Xenograft Versus Silver-Based Dressings in the Management of Partial-Thickness Burn Wounds: A Systematic Review and Meta-Analysis
Source: J Clin Med. 2024 Mar 13;13(6):1642. doi: 10.3390/jcm13061642 (PMC10971678; doi:10.3390/jcm13061642)
Supplement: Supplementary file 1 [file jcm-13-01642-s001.zip › jcm-2804428-supplementary.pdf]

Supplementary Data

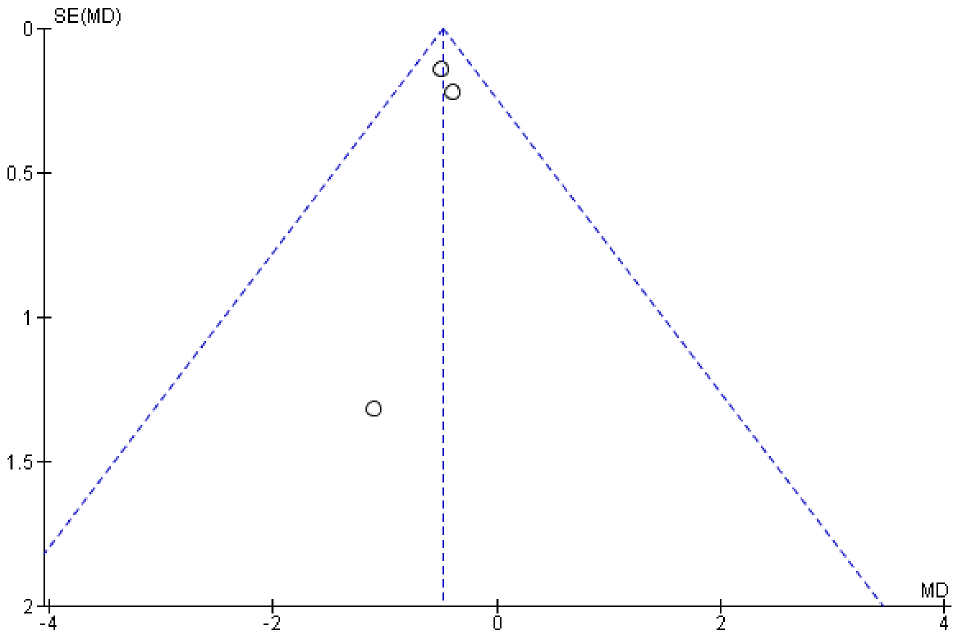

Figure S1. Funnel plot of comparison: 2 Tilapia Skin x silver-based dressings, outcome: 3.2.1 Complete re-epithelialization.

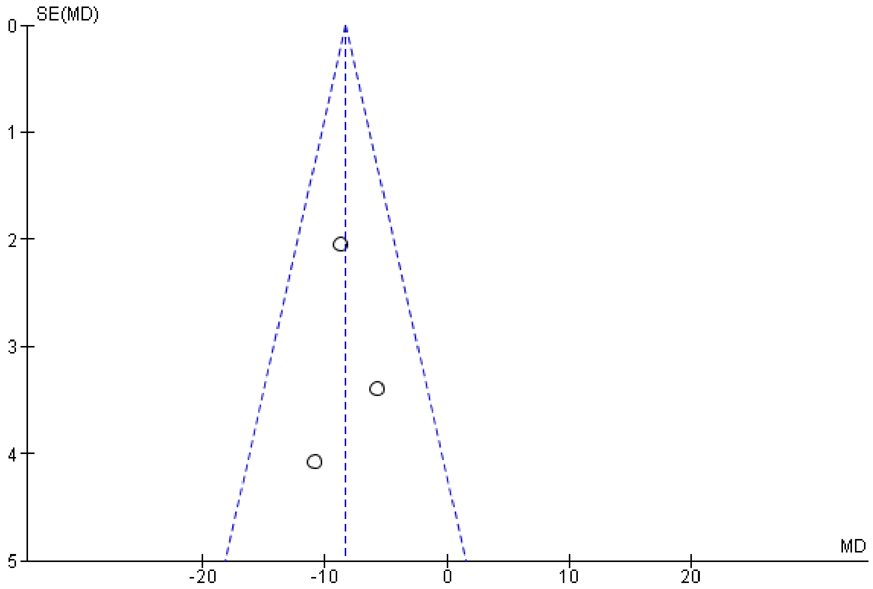

Figure S2. Funnel plot of comparison: 2 Tilapia Skin x silver-based dressings, outcome: 3.2.2 Pain Intensity

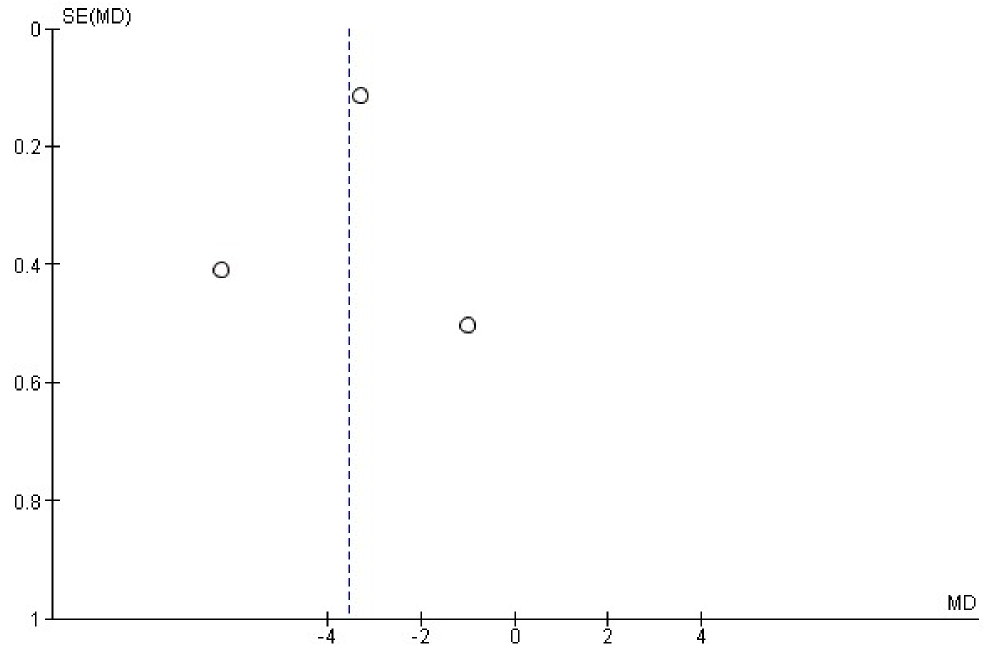

Figure S3. Funnel plot of comparison: 2 Tilapia Skin x silver-based dressings, outcome: 3.2.3 Number of dressings performed.

	Tilapia skin			Silver-based dressings				Mean Difference
Study or Subgroup	Mean	SD	Total	Mean	SD	Total	Weight	IV, Random, 95% CI
Lima 2020	3	0.76	15	9.27	1.39	15	0.0%	-6.27 [-7.07, -5.47]
Lima 2021	1	0.78	12	2	1.56	12	47.7%	-1.00 [-1.99, -0.01]
Lima 2021 (PHASE III)	1.6	0.7	57	4.9	0.5	58	52.3%	-3.30 [-3.52, -3.08]
**Total (95% CI)**			**69**			**70**	**100.0%**	**-2.20 [-4.45, 0.05]**

Heterogeneity: $Tau^2 = 2.51$; $Chi^2 = 19.86$, df = 1 ($P < 0.00001$); $I^2 = 95\%$
Test for overall effect: Z = 1.92 (P = 0.06)

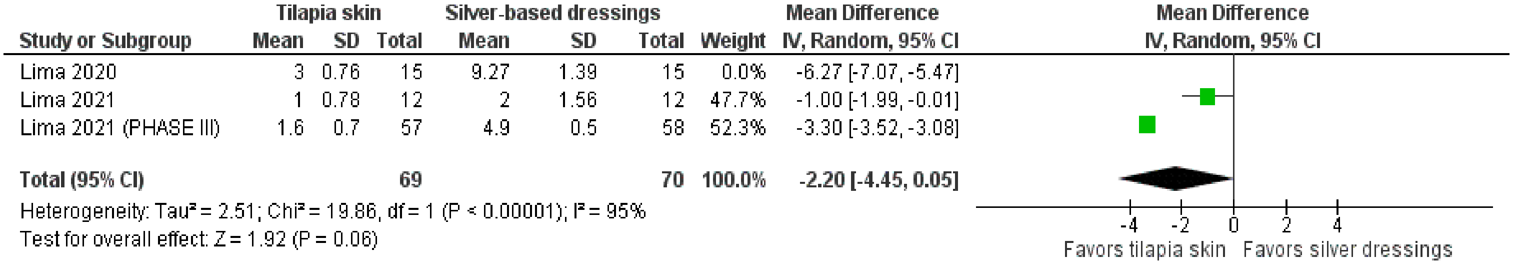

Figure S4. Number of dressings performed leave one out analysis;
